# Blood Groups Distribution and Gene Diversity of the ABO and Rh (D)* Loci* in the Mexican Population

**DOI:** 10.1155/2018/1925619

**Published:** 2018-04-23

**Authors:** Adrián Canizalez-Román, Abraham Campos-Romero, José A. Castro-Sánchez, Mario A. López-Martínez, Francisco J. Andrade-Muñoz, Cinthia K. Cruz-Zamudio, Tania G. Ortíz-Espinoza, Nidia León-Sicairos, Alma M. Gaudrón Llanos, Jorge Velázquez-Román, Héctor Flores-Villaseñor, Secundino Muro-Amador, Jesús J. Martínez-García, Jonathan Alcántar-Fernández

**Affiliations:** ^1^CIASaP, School of Medicine, Autonomous University of Sinaloa, 80246 Culiacan, SIN, Mexico; ^2^The Women's Hospital, Secretariat of Health, 80127 Culiacan, SIN, Mexico; ^3^Innovation and Research Division, Salud Digna para Todos, 80000 Culiacan, SIN, Mexico; ^4^Medical Division, Salud Digna para Todos, 80000 Culiacan, SIN, Mexico; ^5^Clinical Laboratory Division, Salud Digna para Todos, 80000 Culiacan, SIN, Mexico; ^6^Pediatric Hospital of Sinaloa, 80200 Culiacan, SIN, Mexico

## Abstract

**Objective:**

To determine the frequency and distribution of ABO and Rh (D) antigens and, additionally, investigate gene diversity and the structure of Mexican populations.

**Materials and Methods:**

Blood groups were tested in 271,164 subjects from 2014 to 2016. The ABO blood group was determined by agglutination using the antibodies anti-A, Anti-B, and Anti-D for the Rh factor, respectively.

**Results:**

The overall distribution of ABO and Rh (D) groups in the population studied was as follows: O: 61.82%; A: 27.44%; B: 8.93%; and AB: 1.81%. For the Rh group, 95.58% of people were Rh (D), and 4.42% were Rh (d). Different distributions of blood groups across regions were found; additionally, genetic analysis revealed that the *I*^*O*^ and *I*^*D*^ allele showed an increasing trend from the north to the center, while the *I*^*A*^ and *I*^*d*^ allele tended to increase from the center to the north. Also, we found more gene diversity in both* loci* in the north compared with the center, suggesting population structure in Mexico.

**Conclusion:**

This work could help health institutions to identify where they can obtain blood products necessary for medical interventions. Moreover, this piece of information contributes to the knowledge of the genetic structure of the Mexican populations which could have significant implications in different fields of biomedicine.

## 1. Introduction

More than a century has passed since the discovery of ABO system by Karl Landsteiner in 1901; this knowledge has contributed to the understanding of some mechanisms basis of heredity, and today it still has a great conceptual and clinical interest [1]; also, blood antigens had been related to predisposing individuals to some diseases like cancer, diabetes, infectious diseases, and heart illnesses [2–4] or protecting individuals against some diseases such as malaria and diabetes [5, 6]. Moreover, blood antigens had been used to evaluate ethnic diversity of human populations [7], for which they have been widely studied in population genetics [8, 9].

The ABO and Rh blood groups are the most relevant antigens because their incompatibility produces hemolysis [10] and hemolytic disease of the newborn in the case of the Rh group [11]. Furthermore, blood antigens play an important role in the success of transfusions and organ transplants [12]; compatibility of ABO groups between donors and recipients is desirable to avoid immune responses against allograft and reducing the use of immunosuppressive therapies.

The main challenge is to understand how to promote tolerant immune responses against allograft tissues; different factors such as age, viral serology, and gender had been studied to identify their relationship with allograft rejection. Additionally, the role of ethnicity has been poorly studied [13–17]. For the above, molecular biology has taken great importance to identify genetic variants present in several ethnic groups that could play an important role in the success of allograft transplants between donors and recipients [18].

There are few works about population genetics in Mexico [19–21]. The first studies were performed by Lisker and colleagues, in indigenous and mestizo populations by studying several blood antigens [9, 22]; however, few populations were studied and currently there is lack of information about blood groups distribution in the country, whereby it is essential to get this information to help health institutions for the effective management of their blood banks that facilitate transplant medicine practices.

Here we report the distribution of ABO and D antigens in 17 states of the country. Additionally, we used the ABO and D loci as a genetic trait to investigate gene structure of Mexican populations. The above will provide information which would support national programs for blood and organ transplant in Mexico as well as increase the knowledge of Mexican genetics.

## 2. Material and Methods

### 2.1. Study Design

A cross-sectional study was conducted in patients who visited the clinics of Salud Digna para Todos in 17 states of Mexico from 2014 to 2016. The selection of participants was performed using a nonprobabilistic sampling with information on the blood group test. From each people, clinical history was obtained before screening for their demographic data. 271,164 subjects were selected between 0 and 90 years of both genders. Ethical approval was obtained from the Ethical and Research Committee of the Faculty of Medicine, Autonomous University of Sinaloa.

### 2.2. Sample Collection and Blood Groups Determination

From each patient, we got approximately 5 ml of peripheral venous blood with the BD Vacutainer® Blood Collection Tube with EDTA. Tubes were inverted for 8 to 10 times to mix well with the anticoagulant. Blood samples were centrifuged at 1000 to 1500 rpm for 10 min. Erythrocytes were separated for the determination of blood type. ABO blood group was determined from each sample by agglutination using anti-A and Anti-B antibodies (Immucor Inc., Norcross, GA, USA); Rh factor was determined by agglutination using Anti-D antibody (Immucor Inc., Norcross, GA, USA). All assays were performed with the Galileo Echo™ Blood Analyzer (Immucor Inc., Norcross, GA, USA) according to manufacturers' recommendations.

### 2.3. Allelic Frequency and Gene Diversity Analysis

Allele frequencies were estimated according to Bernstein's method (1925) [23] from the phenotypic data; the expected frequency was calculated under the assumption of the Hardy-Weinberg equilibrium from Rh and ABO phenotypes, with the Expected-Maximization (EM) algorithm [24]. Gene diversity was analyzed according to Nei [25]. The Nei genetic distances [26] were calculated based on the gene frequencies data of the ABO and D loci, and a dendrogram was constructed using the neighbor-joining (NJ) clustering procedure with the POPTREEW software [27]. The gene frequencies were used for the Principal Components Analysis (PCA).

### 2.4. Statistical Analysis

Demographic and phenotypic data were analyzed with descriptive statistics; proportions of blood groups are shown as a percentage with 95% CI. The chi-squared test was performed to compare differences between groups and categories. *P* values less than 0.05 were regarded as statistically significant. The Wilson score method without continuity correction was performed to calculate 95% CI. Data were analyzed with the Minitab V17 software (Minitab Inc.).

## 3. Results

### 3.1. Blood Groups Distribution by Age and Gender

**Figure 1:**
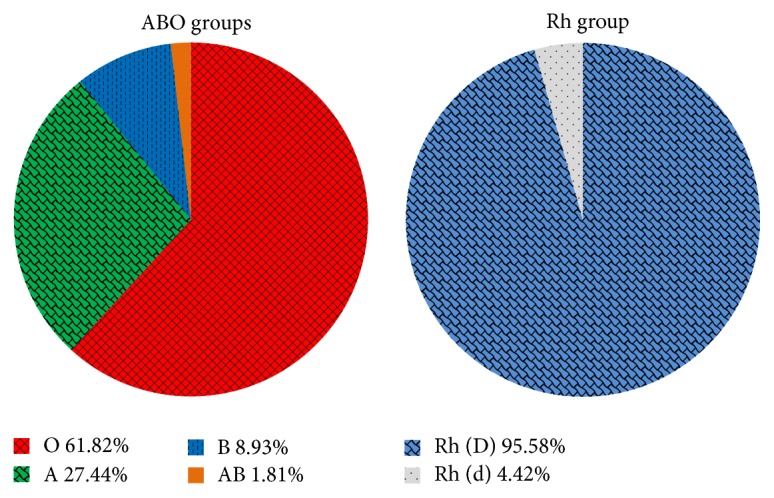
Pie charts summarize ABO and Rh (D) blood groups distribution in Mexico. A total of 271,164 people were tested for blood groups.

The blood types distribution in 271,164 people studied revealed that O was the most frequent (61.82%), followed by A at 27.44% and B at 8.93%, and finally AB group was the less frequent at 1.81%. Moreover, the Rh (D) group was found in 95.58% of the people studied, and 4.42% were identified with the Rh (d) group (Figure 1).

The pooled ABO and Rh groups had the following distribution: the O Rh (D) type was the most frequent (59.26%), followed by A Rh (D) (26.08%), B Rh (D) (8.53%), O Rh (d) (2.56%), AB Rh (D) (1.71%), and A Rh (d) (1.35%). In contrast, B Rh (d) (0.40%) and AB Rh (d) (0.10%) were less frequent which were found in less than 1% of people studied (Table 1).

The distribution of pooled blood antigens among age and gender was analyzed; it was found that they had similar frequencies in people ranges from 0 to 90 years (Table 1). Interestingly, slight differences were observed in some blood types in both genders.

A Rh (D) and AB Rh (D) blood types were more common in males (26.88% and 1.84%, resp.) than females (25.74% and 1.66%, resp.). Meanwhile, O Rh (D) was most prevalent in women (59.78%) than men (58.04%) (Table 1).

### 3.2. Geographic Distribution of Blood Groups

**Figure 2:**
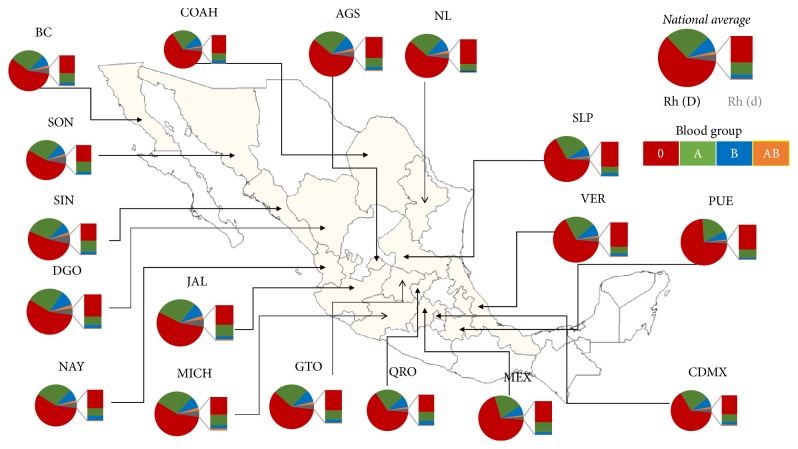
*Map showing the frequency *(%)* of ABO and Rhesus blood groups in different states of Mexico*. Pie charts summarize per-state average proportions of ABO pooled to Rh (D) group; and bars show the proportions of ABO combined with Rh (d) blood group. National average of blood group: 4.42% for Rh (d) (gray color) and 95.58% for Rh (D), involving 61.82% for O (red color), 27.44% for A (green color), 8.93% for B (blue color), and 1.81% for AB (orange color) groups. BC = Baja California; SON = Sonora; SIN = Sinaloa; DGO = Durango; NAY = Nayarit; COAH = Coahuila; JAL = Jalisco; MICH = Michoacan; NL = Nuevo Leon; GTO = Guanajuato; AGS = Aguascalientes; QRO = Queretaro; SLP = San Luis Potosi; VER = Veracruz; MEX = Estado de Mexico; PUE = Puebla; CDMX = Ciudad de Mexico.

Blood groups were studied in 17 states which belong to 6 regions of Mexico; the blood groups O and Rh (D) were the most frequent in all states analyzed. However, their frequencies change across the country (Figure 2). The blood type O Rh (D) was more frequent in Puebla (73.15%), Estado de Mexico (69.32%), and San Luis Potosi (66.18%) compared with Sinaloa (52.73%), Jalisco (54.86%), and Sonora (54.97%). Moreover, A Rh (D) blood type was more frequent in Sinaloa (30.52%), Nayarit (28.60%), and Sonora (28.29%) whereas in Puebla (18.34%), Estado de Mexico (20.48%), and Veracruz (21.34%) it was less prevalent (Figure 2 and Table 2).

B Rh (D) was more frequent in Durango (10.86%), Aguascalientes (9.90%), and Nuevo Leon (9.88%); in contrast, this group was less prevalent in Puebla (5.97%), Estado de Mexico (7.00%), and San Luis Potosi (7.12%). O Rh (d) was more frequent in Sinaloa (3.73%), Sonora (3.61%), and Durango (3.09%) than Puebla (1.21%), San Luis Potosi (1.29%), and Estado de Mexico (1.36%). For AB Rh (D) we observed that it was more frequent in Durango (2.40%), Jalisco (2.24%), and Michoacan (2.22%) and less frequent in Puebla (0.85%), San Luis Potosi (0.97%), and Estado de Mexico (1.02%).

The A Rh(−) blood type was more frequent in Sinaloa (2.41%), Sonora (2.19%), and Jalisco (1.77%) than Puebla (0.39%), San Luis Potosi (0.32%), and Veracruz (0.56%) in which it was less frequent. The B Rh (d) blood type was more frequent in Nayarit (0.63%), Sinaloa (0.62%), and Sonora (0.55%) and was less prevalent in Puebla (0.08%), San Luis Potosi (0.16%), and Estado de Mexico (0.18%); finally, the AB Rh (d) group was more frequent in Michoacan (0.24%), Sonora (0.13%), and Sinaloa (0.12%), was absent in Nuevo Leon (0.00%) and San Luis Potosi (0.00%), and was less frequent in Puebla (0.02%) and Estado de Mexico (0.02%) (Table 2).

### 3.3. Genetic Analysis of the ABO and D* Loci*

#### 3.3.1. Heterozygosity and Hardy-Weinberg Equilibrium at the ABO and D* Loci*

We analyzed the heterozygosity of the ABO and D* loci* in the sample studied (Table 3). The highest heterozygosities of the ABO* locus* were found in Sinaloa (*H* = 0.3949), Jalisco (*H* = 0.3862), and Sonora (*H* = 0.3795), while the lowest one was found in Puebla (*H* = 0.2446), Estado de Mexico (*H* = 0.2777), and San Luis Potosi (*H* = 0.3051). Similarly, for the D* locus* in Sinaloa (*H* = 0.4559), Sonora (*H* = 0.4443), and Durango (*H* = 0.3899) the highest heterozygosities were observed, while the lowest heterozygosities were observed in Puebla (*H* = 0.2431), San Luis Potosi (*H* = 0.2490), and Estado de Mexico (*H* = 0.2735) (Table 3).

According to these observations, populations were analyzed to know if they were in the Hardy-Weinberg equilibrium (HWE). For the ABO* locus*, significant deviations were observed in Jalisco (*χ*^2^ = 6.03; *p* < 0.05) and Ciudad de Mexico (*χ*^2^ = 5.42; *p* < 0.05). In contrast, we found that the* locus* D was in HW equilibrium in all populations analyzed (Table 3).

#### 3.3.2. Allelic Distribution

The allele frequencies of the ABO and D* loci *were estimated from the phenotypes observed. It was found that allele *I*^*A*^ was more frequent in Sinaloa (*I*^*A*^ = 0.1937), Jalisco (*I*^*A*^ = 0.1776), and Sonora (*I*^*A*^ = 0.1773), while in Puebla (*I*^*A*^ = 0.1033), Estado de Mexico (*I*^*A*^ = 0.1177), and Veracruz (*I*^*A*^ = 0.1239) it was less frequent (Table 3). Allele *I*^*B*^ was more frequent in Durango (*I*^*B*^ = 0.0704), Aguascalientes (*I*^*B*^ = 0.0630), and Jalisco (*I*^*B*^ = 0.0618), while in Puebla (*I*^*B*^ = 0.0344), Estado de Mexico (*I*^*B*^ = 0.0417), and San Luis Potosi (*I*^*B*^ = 0.0432) this allele was less frequent (Table 3).

*I* ^*O*^ was more frequent in Puebla (*I*^*O*^ = 0.8623), Estado de Mexico (*I*^*O*^ = 0.8407), and San Luis Potosi (*I*^*O*^ = 0.8214), while this allele was less frequent in Sinaloa (*I*^*O*^ = 0.7514), Jalisco (*I*^*O*^ = 0.7606), and Sonora (*I*^*O*^ = 0.7653) (Table 3).

For the Rhesus group, the *I*^*D*^ allele was more frequent in Puebla (*I*^*D*^ = 0.8700), San Luis Potosi (*I*^*D*^ = 0.8666), and Estado de Mexico (*I*^*D*^ = 0.8523). In Sinaloa (*I*^*D*^ = 0.7377), Sonora (*I*^*D*^ = 0.7455), and Jalisco (*I*^*D*^ = 0.7674) this allele was less frequent. The *I*^*d*^ allele was more frequent in Sinaloa (*I*^*d*^ = 0.2623), Sonora (*I*^*d*^ = 0.2545), and Jalisco (*I*^*d*^ = 0.2326), while Puebla (*I*^*d*^ = 0.1300), San Luis Potosi (*I*^*d*^ = 0.1334), and Estado de Mexico (*I*^*d*^ = 0.1477) were the states in which this allele was less frequent (Table 3).

**Figure 3:**
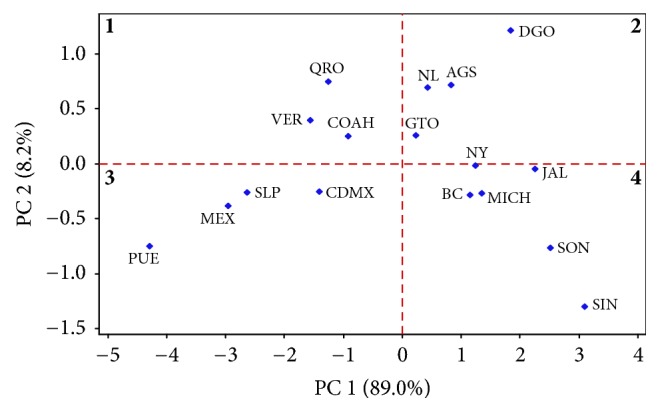
*Principal Component Analysis (PCA) of the ABO and D alleles*. Samples were clustered into four groups according to PC1 and PC2 which explains 97.2% of the variability among populations; groups one and two do not exhibit geographical clustering; however, groups three and four show regional clustering in the south-center and east (group 3) and west and northwest (group 4). BC = Baja California; SON = Sonora; SIN = Sinaloa; DGO = Durango; NAY = Nayarit; COAH = Coahuila; JAL = Jalisco; MICH = Michoacan; NL = Nuevo Leon; GTO = Guanajuato; AGS = Aguascalientes; QRO = Queretaro; SLP = San Luis Potosi; VER = Veracruz; MEX = Estado de Mexico; PUE = Puebla; CDMX = Ciudad de Mexico.

The ABO and D* loci* were not distributed homogeneously among states; to understand the variation observed we used the Principal Component Analysis (PCA) based on the allele frequencies of the ABO and D* loci* (Table 3). PC1 and PC2 explain 97.2% of the total variation of the ABO and Rh blood groups distribution. The PC1 differentiates populations with high frequencies of *I*^*A*^, *I*^*B*^, and *I*^*d*^ alleles; meanwhile, PC2 separates those with high proportions of *I*^*B*^ and *I*^*D*^ alleles; according to this, four groups could be defined (Figure 3).

The first group includes the states of Coahuila, Queretaro, and Veracruz which have moderate frequencies of the *I*^*B*^ and *I*^*d*^ alleles (first quadrant). The second comprises Durango, Aguascalientes, Nuevo Leon, and Guanajuato which have higher proportions of the* I*^*B*^ and* I*^*D *^alleles and moderate frequencies of *I*^*A*^ allele (second quadrant).

Both groups have states with higher frequencies of the B Rh (D) and B Rh (d) blood types; in the second group, there are states with moderate proportions of the AB blood type. A geographic clustering in these groups was not evident (Figure 3).

Interestingly, in the third and fourth group, a geographical clustering was observed; the third group includes the states of Puebla, San Luis Potosi, Estado de Mexico, and Ciudad de Mexico (third quadrant) which have higher frequencies of *I*^*O*^ and *I*^*D*^ alleles and lower frequencies of the *I*^*A*^ and *I*^*B*^ alleles. These states are located in the east, north-center, and south-center of the country (Table 3). The fourth group has higher frequencies of *I*^*A*^ and *I*^*d*^ alleles and includes the states of Sinaloa, Sonora, Baja California, Michoacan, Jalisco, and Nayarit which belong to northwest and west of Mexico (Table 3).

**Figure 4:**
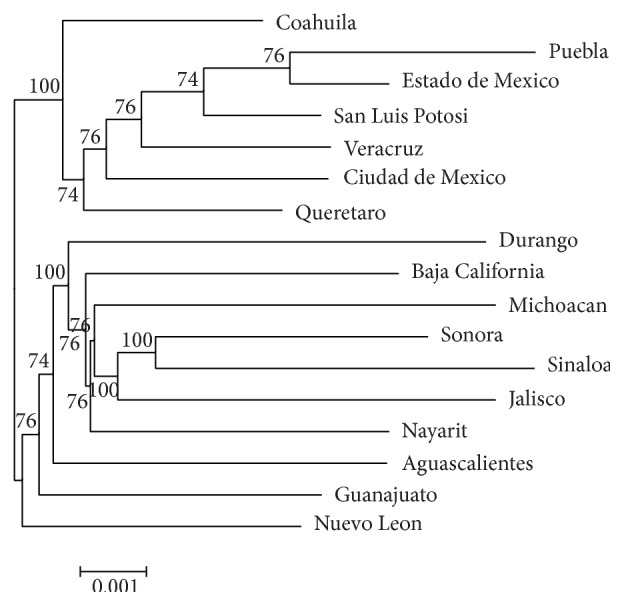
Neighbor-joining phylogenetic tree of ABO and D antigens in Mexican populations. Genetic distances were estimated using the Nei method. Clustering of populations was tested with 1000 bootstrap replicates; the numbers at the nodes are the bootstrap values.

We used the neighbor-joining (NJ) clustering procedure based on Nei's genetic distances (DA) to analyze the relationship between populations studied. Two main clusters were identified; the first includes the states of Puebla, Estado de Mexico, San Luis Potosi, Ciudad de Mexico, Veracruz, Queretaro, and Coahuila (which have higher frequencies of the *I*^*O*^ allele; Table 3). In the second; Sinaloa, Sonora, Jalisco, Michoacan, Nayarit, Baja California, and Durango were included (which have higher frequencies of the *I*^*A*^ and *I*^*B*^ alleles in the case of Durango). The states of Aguascalientes, Guanajuato, and Nuevo Leon, also, were included in this group, since they have higher frequencies of the *I*^*B*^ allele and are more related to Durango than the other states of this group (Figure 4).

#### 3.3.3. Population Structure and Gene Diversity

The results of the PCA and the NJ clustering of the ABO and D allele's frequencies evidence gene diversity among Mexican populations. To formally measure the genetic differentiation (*G*_*ST*_), we cluster all population studied in 4 main regions and performed analysis for each* locus* and pooled* loci*. Groups are north (Baja California, Sonora, Sinaloa, Nuevo Leon, Durango, and Coahuila), west (Nayarit, Jalisco, and Michoacan), east (Puebla and Veracruz), and center (San Luis Potosi, Aguascalientes, Guanajuato, Queretaro, Estado de Mexico, and Ciudad de Mexico).

The overall gene diversity was higher at the ABO* locus *(*H*_*T*_ = 0.3536) than the D* locus *(*H*_*T*_ = 0.3320); similarly, the gene diversity within populations was higher in the ABO* locus *(*H*_*S*_ = 0.3411) than the D* locus *(*H*_*S*_ = 0.3093). However, gene differentiation (*G*_*ST*_) was higher in the D* locus *(*G*_*ST*_ = 0.0686) than the ABO* locus *(*G*_*ST*_ = 0.0353) (Table 4).

The regional analysis shows that the highest gene diversity (*H*_*T*_) and variability within populations (*H*_*S*_) for the ABO* locus* were found in the west (*H*_*T*_ = 0.3839; *H*_*S*_ = 0.3791); meanwhile the lowest was observed in the east (*H*_*T*_ = 0.2580, *H*_*S*_ = 0.2741). For the D* locus, *the highest gene diversity and variability within the population were observed in the north (*H*_*T*_ = 0.3556, *H*_*S*_ = 0.3435), and the lowest one was found in the east (*H*_*T*_ = 0.2409, *H*_*S*_ = 0.2545) (Table 4).

The highest genetic differentiation for the ABO* locus* was found in the north (*G*_*ST*_ = 0.0161) and in the west (*G*_*ST*_ = 0.0361) for the D* locus. *Surprisingly, a negative value for the genetic differentiation parameter (*G*_*ST*_) in the east was found, suggesting no differentiation in both* loci* in this region, which is consistent with low heterozygosities observed (Table 4).

## 4. Discussion

The study of blood groups is fundamental in the clinical practice due to the inherent relationship in transfusion medicine and organ transplants [12]. In Mexico, the rate of blood donations in 2014 increased from 15.66 per 1000 individuals to 17.33 per 1000 individuals in 2015 [28]. The above is due to the improvement in donor blood programs established in the country; however, in blood banks it is challenging to get enough blood units, especially for the less frequent blood types.

For the above, it is necessary to implement effective programs among health institutions to get specific blood types and products according to their geographic distribution. However, the information about the proportions of the ABO and Rh (D) blood groups in Mexico is insufficient; to meet this need here we report the distribution of ABO and Rh (D) blood groups in several areas of the country.

To our knowledge, this is the first multicenter study of the ABO and Rh (D) blood groups in Mexico, in which the overall distribution in both genders, in a wide age range, and in different states of the country has been analyzed. A total of 271,164 individuals from 17 states of Mexico were studied between the years 2014 and 2016. We found that the ABO groups distribution was O (61.82%), A (27.44%), B (8.93%), and AB (1.81%). Our observations were similar to previous reports in which the O group was the most frequent, followed by the A, B, and AB groups [29–35].

The frequencies of the ABO antigens in Mexican populations are different from those observed in other Latin American countries like Argentina, Bolivia, Brazil, and Dominican Republic [36]. Interestingly, the Rh (D) antigen was more frequent in Mexico (95.58%) than what is observed in other Latin American countries [36]. The frequency observed was slightly similar to those found in indigenous populations [37–39], reflecting the complex processes of the admixture giving rise to Mexican mestizo populations [9].

It was found that the frequencies of blood groups were similar among ages; however, slight differences between genders were observed in the A Rh (D), AB Rh (D), and O Rh (D) blood types. The above could be explained by the sampling method used, which would result in the overrepresentation of females in the sample.

Previous studies have been conducted in Mexico to determine the local distribution of the ABO and Rh (D) blood groups; a few of those works were performed in indigenous people [37, 40–42] and the majority in mestizos [29–35]. For this study, samples were obtained from metropolitan cities, most of which are composed of mestizo individuals; variability in proportions of blood antigens was found in different areas of the country. The frequencies observed in Coahuila, Nuevo Leon, Jalisco, and Ciudad de Mexico were similar to that previously reported [30–34]; however, for Durango, Puebla, and Guanajuato, proportions of blood antigens were different compared with our results [29, 31, 34, 35]. Moreover, the allele frequencies for both* loci* in previous works were different from those reported here. Additionally, populations studied in those reports were not in Hardy-Weinberg genetic equilibrium (HWE) in both* loci* [30–32, 34, 35] except in Puebla [29] and Coahuila [31].

Samples analyzed in this work were in HWE for the ABO* locus* except those coming from Jalisco and Ciudad de Mexico. The above could result from nonrandom sampling or internal migrations (that happens in this states by their socioeconomic development) because the sample size is big and other disturbance events have not been reported in these populations (i.e., inbreeding and mutations). Interestingly, we found that the Rh (D)* locus *was in HWE; however, more studies are needed to corroborate our observations.

The above is important because if populations are in HWE this means that the observed frequencies of blood groups will be similar in each generation. This information will allow health institutions to obtain enough blood units since the site where it is more frequent to get a specific blood type with the confidence that these frequencies will be relatively constant is known, and it will be not necessary to investigate the distribution of blood groups in these populations again as soon.

Additionally, geographical cline of the ABO and D* loci *with remarkably high frequencies in the north and the center for the *I*^*A*^* and I*^*D*^, respectively, was identified; more studies are needed to explain the possible causes underlying these cline distributions in the country. Different factors like migrations, nonrandom mating, and infectious diseases among others would confer evolutionary constraints over this genetic trait [4, 43, 44]; it would be possible that both* loci* have some selection pressure resulting in their current distribution in Mexico; however, this remains unexplored yet.

In this report, we evidenced regional differences of the blood groups distribution; we suspect that these differences could be a result of differentiation between regions; according to this, we studied the genetic structure of the population by using the ABO and D* loci *as genetic markers. Differentiation in Mexican populations was found among regions analyzed; also a higher heterozygosity and gene diversity were observed in the north and west; meanwhile, in the east and south-center we found low heterozygosity and gene diversity.

Despite the wide distribution of the ABO and D/d alleles, the estimation of interpopulation comparison (*G*_*ST*_ and *D*_*ST*_) also evidences genetic differentiation between populations. It is interesting to note that in the east there was no genetic differentiation for both* loci* which was evident by the negative value of the genetic differentiation (*G*_*ST*_) estimator [45]. The above would be possible by the lowest heterozygosity found in Puebla in which the highest frequencies of the *I*^*O*^ and *I*^*D*^ alleles were observed.

It would be interesting to investigate the reason for the reduction in heterozygosity of both* loci* in Puebla. Additionally, it is necessary to sample other populations of the east to corroborate our observations and extend this study to other regions of Mexico to know the countrywide distribution of the ABO and Rh (D) blood groups.

There are a few works about gene diversity in Mexico; our results with the ABO and D* loci* as a genetic trait are consistent with them in which the genetic structure of indigenous and mestizo populations was explored with SNPs as genetic markers [19, 20]. Similar to ours, these works reported that populations in the north have higher heterozygosities with respect to those located in the center and the south of the country [20]. Additionally, they found genetic stratification in indigenous communities [19, 20].

Interestingly, this Native-American population substructure is recapitulated in the genomes of Mexican mestizos [19] which is consistent with our observations of genetic differentiation in Mexican populations across several regions of the country. It is important to take into account the fact that Mexicans are a mestizo population recently established, composed of the admixture of European, African, and majorly Amerindians [19, 20] where the *I*^*O*^ allele is nearly fixed [37, 39, 46]. The above could explain the high frequencies of the *I*^*O*^ allele in Mexico, especially in Puebla in which the Amerindian ancestry is more prevalent [29, 47] supporting our observations of low heterozygosity, suggesting low admixture in this population.

Currently, there are 68 indigenous groups in Mexico [48] which have their own cultural and economic systems that differ significantly from mestizo populations; these people represent about 6.4% of the entire population [49]. Ruben Lisker performed the first works of Mexican genetics in indigenous populations in the 1960s [9, 22], in which he tried to know the degree of admixture as well as the main ancestral components present in these populations. Recently, some studies have been carried out at the molecular level with the aim of knowing the underlying relationships between indigenous and mestizos [19–21], to reconstruct the history of the Amerindian populations in the continent [50] and their development throughout the country [21]. Additionally, these works have explored the possible effects of the genetic content in the clinic context [19].

At this point, our work contributes to the knowledge of the gene diversity in Mexico by evidencing regional and geographic differentiation into the country. Also, we studied some populations that had not been previously analyzed, thus increasing the information of the population genetics in Mexico.

Here we show that people of the western part (including northwest populations) have a close genetic relationship between them; similarly, populations of the south-center are more related to eastern part; interestingly, east populations kept a distant genetic relationship with western ones. It would be interesting to analyze if there is any influence of gene diversity in clinical traits.

Previous work showed the impact of genetic variation in the accuracy of lung function assessment [19]; it was reported that healthy people with genetic variants common in the east of Mexico had different results on the lung function test than did people from the west [19]. The above suggests that the same criteria to diagnose lung disease could not be applied in both populations because this would result in a misdiagnosis [19]. Additionally, other works have related genetic ancestry in Mexico to susceptibility to breast cancer [51] and diabetes [52]. Together these works show the effects of gene diversity on diagnostic tools and the risk to get some diseases that will have to be taken into account in the future to improve accuracy in biomedicine. Therefore, it is crucial to develop genomic medicine to impact on Mexico's public health positively.

In transplant medicine, several works have studied the effects of genetic variants of a wide range of proteins including Human Leukocyte Antigens (HLA) in the risk of rejection in allograft transplants [15–17, 53, 54]. For example, in Mexico, some works have found a positive association between specific HLA haplotypes and acute kidney rejection [15, 17]. Interestingly, those immunogenic variants are widely distributed among indigenous and mestizo people [47, 55].

For the above, it would be possible to think that gene diversity could play an important role in transplant medicine; in that case, genetically related populations could have lower organ-rejection rate than those with greater genetic distance. Therefore the knowledge of gene diversity could help to select suitable donors and estimate the success of organ transplants as well as the effectiveness of the immunosuppressive therapies to prevent acute rejections; nevertheless, this remains unexplored yet.

This work has some limitations including the sampling method and the indirect determination of the ABO and D allele's frequencies; however, the large sample size and the uniformity in the blood group test ensure the results obtained, which provides a unique opportunity to estimate the blood groups distribution in Mexico. Likewise, we expected that this study helps in the establishment of regional and national programs for blood transfusions and organ transplants according to the distribution of blood antigens. Additionally, our results about gene diversity in 17 states of Mexico will expand the knowledge of anthropology of the country which will allow understanding the establishment of the current Mexican population and their relationship with different ethnic groups around the country.

## 5. Conclusions

This work will provide useful information for health institutions in the establishment of regional and national programs that speed up tissue transplants and blood transfusions needed in clinical practice. Likewise, it will contribute to the study of Mexican genetics by showing its differentiation among the country, which could have important implications in different fields of biomedicine such as transplant medicine and immunology, as well as the treatment and diagnosis of several pathologies present in the country. Additionally, this work is expected to generate deep interest in ethnologists and anthropologists related to the study of population' genetics in Mexico, as well as physicians interested in the application of the molecular genetics in diagnosis and clinical practice.

## Figures and Tables

**Table 1:** Distribution of ABO and Rhesus blood groups by age and gender.

Parameters	*n*	Blood groups % (95% CI)							
		A		B		AB		O	
		Rh (D)	Rh (d)	Rh (D)	Rh (d)	Rh (D)	Rh (d)	Rh (D)	Rh (d)
Age									
0–9	27,173	26.97 (26.45–27.50)	1.52 (1.38–1.67)	9.16 (8.82–9.50)	0.45 (0.37–0.53)	1.84 (1.68–2.00)	0.07 (0.04–0.11)	57.37 (56.78–57.96)	2.63 (2.44–2.82)
10–19	60,297	25.80 (25.45–26.15)	1.17 (1.09–1.26)	8.51 (8.29–8.74)	0.41 (0.36–0.46)	1.65 (1.55–1.76)	0.07 (0.05–0.09)	59.97 (59.98–60.36)	2.42 (2.30–2.54)
20–29	101,507	25.40 (25.13–25.67)	1.28 (1.22–1.36)	8.26 (8.10–8.44)	0.36 (0.33–0.40)	1.62 (1.54–1.70)	0.11 (0.09–0.13)	60.44 (60.14–60.74)	2.52 (2.43–2.62)
30–39	43,134	26.15 (25.74–26.57)	1.46 (1.35–1.58)	8.25 (8.00–8.52)	0.39 (0.33–0.45)	1.69 (1.58–1.82)	0.11 (0.08–0.14)	59.29 (58.83–59.75)	2.65 (2.50–2.80)
40–49	17,065	27.15 (26.49–27.82)	1.42 (1.26–1.61)	9.21 (8.79–9.65)	0.44 (0.35–0.55)	1.88 (1.68–2.09)	0.10 (0.06–0.16)	57.06 (56.31–57.80)	2.74 (2.51–3.00)
50–59	10,085	28.60 (27.72–29.49)	1.57 (1.34–1.83)	8.90 (8.36–9.48)	0.58 (0.45–0.74)	2.00 (1.75–2.30)	0.13 (0.08–0.22)	57.54 (54.57–56.51)	2.69 (2.39–3.02)
60–69	6,859	27.69 (26.64–28.76)	1.71 (1.43–2.04)	9.36 (8.69–10.07)	0.54 (0.39–0.74)	2.19 (1.87–2.56)	0.15 (0.08–0.27)	55.59 (54.41–56.76)	2.78 (2.42–3.20)
70–79	3,742	27.53 (26.12–28.98)	1.79 (1.41–2.27)	9.25 (8.36–10.22)	0.53 (0.35–0.82)	2.08 (1.67–2.59)	0.16 (0.07–0.35)	55.72 (54.12–57.30)	2.94 (2.44–3.53)
80–90	1,302	25.81 (23.50–28.25)	2.23 (1.56–3.18)	7.37 (6.08–8.92)	0.46 (0.21–1.00)	2.07 (1.43–3.00)	0.08 (0.01–0.43)	59.14 (56.45–61.78)	2.84 (2.07–3.89)
Gender									
Female	189,482	25.74 (25.54–25.94)	1.33 (1.28–1.39)	8.48 (8.36–8.61)	0.40 (0.37–0.43)	1.66 (1.60–1.72)	0.09 (0.08–0.11)	59.78 (59.56–60.00)	2.52 (2.45–2.59)
Male	81,682	26.88 (26.58–27.19)	1.40 (1.32–1.48)	8.64 (8.45–8.83)	0.43 (0.38–0.47)	1.84 (1.75–1.94)	0.11 (0.09–0.14)	58.04 (57.70–58.38)	2.67 (2.56–2.78)

*Total*	271,164	26.08 (25.92–26.25)	1.35 (1.31–1.40)	8.53 (8.42–8.63)	0.40 (0.38–0.43)	1.71 (1.67–1.76)	0.10 (0.09–0.11)	59.26 (59.07–59.44)	2.56 (2.50–2.62)

**Table 2:** Geographic distribution of ABO and Rh blood groups.

Region	State	*n*	Blood groups% (95% CI)							
			A		B		AB		O	
			Rh (D)	Rh (d)	Rh (D)	Rh (d)	Rh (D)	Rh (d)	Rh (D)	Rh (d)
Northwest	Baja California	45,716	27.36 (26.95–27.77)	1.43 (1.33–1.55)	8.64 (8.39–8.90)	0.39 (0.34–0.46)	1.82 (1.70–1.94)	0.10 (0.08–0.14)	57.58 (57.13–58.03)	2.67 (2.52–2.82)
	Sonora	14,479	28.29 (27.56–29.03)	2.19 (1.96–2.44)	8.56 (8.11–9.02)	0.55 (0.44–0.69)	1.71 (1.51–1.93)	0.13 (0.08–0.20)	54.97 (54.16–55.78)	3.61 (3.31–3.92)
	Sinaloa	40,449	30.52 (30.07–30.97)	2.41 (2.26–2.56)	7.94 (7.68–8.20)	0.62 (0.55–0.70)	1.93 (1.81–2.07)	0.12 (0.09–0.16)	52.73 (52.25–53.22)	3.73 (3.55–3.92)
	Durango	4,925	25.75 (24.54–26.99)	1.14 (0.88–1.47)	10.86 (10.02–11.76)	0.47 (0.31–0.70)	2.40 (2.00–2.86)	0.10 (0.04–0.24)	56.20 (54.81–57.58)	3.09 (2.64–3.61)

Northeast	Coahuila	19,823	22.67 (22.09–23.26)	0.82 (0.70–0.95)	8.68 (8.30–9.08)	0.33 (0.26–0.42)	1.28 (1.13–1.44)	0.05 (0.03–0.09)	64.05 (63.38–64.71)	2.12 (1.93–2.33)
	Nuevo Leon	921	24.86 (22.18–27.76)	0.76 (0.37–1.56)	9.88 (8.12–11.98)	0.22 (0.06–0.79)	1.85 (1.15–2.93)	0.00 (0.00–0.41)	59.50 (56.30–62.63)	2.93 (2.02–4.23)

West	Nayarit	5,270	28.60 (27.39–29.83)	1.02 (0.79–1.33)	8.69 (7.96–9.48)	0.63 (0.45–0.88)	1.76 (1.44–2.15)	0.09 (0.04–0.22)	56.70 (55.36–58.03)	2.50 (2.11–2.96)
	Jalisco	29,206	28.18 (27.67–28.70)	1.77 (1.62–1.92)	9.31 (8.98–9.65)	0.47 (0.40–0.56)	2.24 (2.07–2.41)	0.18 (0.14–0.24)	54.86 (54.29–55.43)	2.99 (2.80–3.19)
	Michoacan	2,477	28.10 (26.36–29.90)	1.41 (1.02–1.95)	8.60 (7.56–9.77)	0.44 (0.25–0.79)	2.22 (1.71–2.88)	0.24 (0.11–0.53)	56.64 (54.68–58.58)	2.34 (1.81–3.01)

East	Puebla	6,212	18.34 (17.40–19.32)	0.39 (0.26–0.57)	5.97 (5.41–6.59)	0.08 (0.03–0.19)	0.85 (0.65–1.11)	0.02 (0.00–0.09)	73.15 (72.03–74.23)	1.21 (0.96–1.51)
	Veracruz	1,790	21.34 (19.51–23.30)	0.56 (0.30–1.02)	8.72 (7.50–10.11)	0.22 (0.09–0.57)	1.28 (0.86–1.92)	0.06 (0.01–0.32)	65.75 (63.52–67.92)	2.07 (1.50–2.83)

North-center	San Luis Potosi	618	23.95 (20.75–27.47)	0.32 (0.09–1.17)	7.12 (5.35–9.42)	0.16 (0.03–0.91)	0.97 (0.45–2.10)	0.00 (0.00–0.62)	66.18 (62.36–69.80)	1.29 (0.66–2.53)
	Aguascalientes	11,709	25.13 (24.36–25.93)	1.12 (0.94–1.33)	9.90 (9.37–10.45)	0.38 (0.28–0.50)	1.96 (1.72–2.22)	0.09 (0.05–0.17)	58.87 (57.98–59.76)	2.55 (2.28–2.86)
	Guanajuato	41,648	25.85 (25.43–26.27)	0.98 (0.89–1.08)	8.98 (8.71–9.26)	0.35 (0.30–0.41)	1.78 (1.65–1.91)	0.07 (0.05–0.11)	59.78 (59.31–60.25)	2.20 (2.07–2.35)
	Queretaro	9,402	22.86 (22.03–23.72)	0.74 (0.59–0.94)	9.04 (8.48–9.64)	0.36 (0.26–0.50)	1.23 (1.03–1.48)	0.05 (0.02–0.12)	64.26 (63.29–65.22)	1.45 (1.22–1.71)

South-center	Estado de Mexico	22,275	20.48 (19.95–21.01)	0.63 (0.53–0.74)	7.00 (6.68–7.35)	0.18 (0.13–0.24)	1.02 (0.90–1.16)	0.02 (0.01–0.05)	69.32 (68.71–69.92)	1.36 (1.21–1.52)
	Ciudad de Mexico	14,244	22.96 (22.28–23.66 )	0.74 (0.61–0.89)	7.76 (7.34–8.21)	0.28 (0.21–0.38)	1.43 (1.24–1.63)	0.11 (0.06–0.17)	64.87 (64.08–65.65)	1.85 (1.64–2.09)

*Total*		271,164	26.08 (25.92–26.25)	1.35 (1.31–1.40)	8.53 (8.42–8.63)	0.40 (0.38–0.43)	1.71 (1.67–1.76)	0.10 (0.09–0.11)	59.26 (59.07–59.44)	2.56 (2.50–2.62)

**Table 3:** Analysis of allele frequencies at the ABO and D *loci* and Hardy-Weinberg equilibrium.

Region	State	ABO *locus*			HW statistics		*Rh locus*		HW statistics		Heterozygosity (*H*)	
		*I* ^*A*^	*I* ^*B*^	*I* ^*O*^	*χ* ^2^	*p*	*I* ^*d*^	*I* ^*D*^	*χ* ^2^	*p*	ABO	D
Northwest	Baja California	0.1676	0.0562	0.7762	0.29	>0.1	0.2145	0.7855	0.09	>0.1	0.3663	0.3829
	Sonora	0.1773	0.0574	0.7653	2.45	>0.1	0.2545	0.7455	0.39	>0.1	0.3795	0.4443
	Sinaloa	0.1937	0.0549	0.7514	0.84	>0.1	0.2623	0.7377	0.60	>0.1	0.3949	0.4559
	Durango	0.1596	0.0704	0.7700	1.23	>0.1	0.2189	0.7811	0.45	>0.1	0.3766	0.3899

Northeast	Coahuila	0.1329	0.0536	0.8135	1.26	>0.1	0.1823	0.8177	0.33	>0.1	0.3178	0.3314
	Nuevo Leon	0.1484	0.0615	0.7901	0.01	>0.1	0.1977	0.8023	0.39	>0.1	0.3495	0.3562

West	Nayarit	0.1722	0.0583	0.7694	0.52	>0.1	0.2062	0.7938	0.28	>0.1	0.3748	0.3697
	Jalisco	0.1776	0.0618	0.7606	**6.03**	**<0.05**	0.2326	0.7674	1.90	>0.1	0.3862	0.4110
	Michoacan	0.1752	0.0568	0.7680	2.34	>0.1	0.2107	0.7893	0.66	>0.1	0.3764	0.3767

East	Puebla	0.1033	0.0344	0.8623	1.99	>0.1	0.1300	0.8700	1.60	>0.1	0.2446	0.2431
	Veracruz	0.1239	0.0526	0.8235	0.02	>0.1	0.1704	0.8296	0.68	>0.1	0.3035	0.3118

North-center	San Luis Potosi	0.1354	0.0432	0.8214	0.19	>0.1	0.1334	0.8666	0.10	>0.1	0.3051	0.2490
	Aguascalientes	0.1533	0.0630	0.7837	0.76	>0.1	0.2035	0.7965	0.85	>0.1	0.3583	0.3656
	Guanajuato	0.1555	0.0572	0.7873	1.10	>0.1	0.1900	0.8100	1.40	>0.1	0.3527	0.3441
	Queretaro	0.1333	0.0561	0.8106	2.54	>0.1	0.1614	0.8386	1.80	>0.1	0.3220	0.2968

South-center	Estado de Mexico	0.1177	0.0417	0.8407	0.76	>0.1	0.1477	0.8523	0.89	>0.1	0.2777	0.2735
	Ciudad de Mexico	0.1353	0.0478	0.8168	**5.42**	**<0.05**	0.1725	0.8275	2.20	>0.1	0.3122	0.3153

**Table 4:** Analysis of gene diversity for the ABO and D *loci* in Mexican populations.

Population	*Locus*	*H*_*S*_	*H*_*T*_	*D*_*ST*_	*G*_*ST*_
North	ABO	0.3642	0.3701	0.0060	0.0161
	Rh (D)	0.3435	0.3556	0.0121	0.0341
	*Pooled*	0.3539	0.3629	0.0091	0.0252

East	ABO	0.2741	0.2580	−0.0162	−0.0625
	Rh (D)	0.2545	0.2409	−0.0136	−0.0564
	*Pooled*	0.2643	0.2495	−0.0149	−0.0595

West	ABO	0.3791	0.3839	0.0048	0.0125
	Rh (D)	0.3390	0.3516	0.0126	0.0361
	*Pooled*	0.3591	0.3678	0.0087	0.0243

Center	ABO	0.3213	0.3280	0.0067	0.0204
	Rh (D)	0.2786	0.2923	0.0137	0.0470
	*Pooled*	0.3000	0.3102	0.0102	0.0337

All (4 regions)	ABO	0.3411	0.3536	0.0125	0.0353
	Rh (D)	0.3093	0.3320	0.0227	0.0686
	*Pooled*	0.3252	0.3428	0.0176	0.0520

*H*_*S*_: genetic diversity within populations; *H*_*T*_: total genetic diversity; *D*_*ST*_: genetic diversity among populations; *G*_*ST*_: genetic differentiation.
